# Minimally invasive esophagectomy and radical lymph node dissection without recurrent laryngeal nerve paralysis

**DOI:** 10.1007/s00464-020-07372-3

**Published:** 2020-02-03

**Authors:** Koji Otsuka, Masahiko Murakami, Satoru Goto, Tomotake Ariyoshi, Takeshi Yamashita, Akira Saito, Masahiro Kohmoto, Rei Kato, Alan Kawarai Lefor, Takeshi Aoki

**Affiliations:** 1 Department of Surgery, Division of Gastroenterological and General Surgery, School of Medicine, Showa University, 1-5-8 Hatanodai, Shinagawa-ku, Tokyo, 142-8666 Japan; 2 Department of Surgery, Jichi Medical University, Tochigi, Japan

**Keywords:** Minimally invasive esophagectomy, Radical lymphadenectomy, Recurrent laryngeal nerve paralysis, Prevent complications, Native tissue preservation, Micro-anatomical layer

## Abstract

**Background:**

We introduce a novel operative technique to dissect lymph nodes adjacent to the recurrent laryngeal nerve, referred to as the “native tissue preservation” technique. Using this technique, there was no damage to the recurrent laryngeal nerve, which is maintained in its anatomical position.

**Methods:**

From September 2016 to December 2018, minimally invasive esophagectomy was performed in the left lateral decubitus position in 87 patients with esophageal cancer. The native tissue preservation technique for lymphadenectomy around the recurrent laryngeal nerve was used, and all patients were evaluated for recurrent laryngeal nerve paralysis.

**Results:**

Minimally invasive esophagectomy was completed in all patients without conversion to thoracotomy. Although an extended lymphadenectomy was performed in all patients, there were no grade II or higher complications (Clavien–Dindo classification) and no incidence of recurrent laryngeal nerve paralysis.

**Conclusion:**

The native tissue preservation technique may reduce the incidence of recurrent laryngeal nerve paralysis after minimally invasive esophagectomy with radical lymph node dissection.

**Electronic supplementary material:**

The online version of this article (10.1007/s00464-020-07372-3) contains supplementary material, which is available to authorized users.

Recurrent laryngeal nerve paralysis (RLNP) causes incomplete closure of the vocal cord folds and limits the ability to successfully cough, as the patient cannot develop sufficient pressure. The risk of pulmonary complications is greatly increased in patients with RLNP because of an increased likelihood of aspiration and the development of aspiration pneumonia [1]. RLNP is considered a major postoperative complication of esophageal surgery. Although it depends on the extent of lymph node dissection, the reported incidence of RLNP after esophagectomy varies from 8.3 to 40.9% [2–4].

We previously reported our procedure for thoracoscopic esophagectomy in the left lateral decubitus position and suggested the benefits of minimally invasive esophagectomy and use of the technique to limit morbidity [5]. Intraoperative nerve injury is caused by thermal damage, stretching, cutting, compression, and vascular compromise to the nerve. These mechanisms more frequently affect the left recurrent laryngeal nerve compared to the right, because of its longer course and vulnerability within the mediastinum. Minimally invasive esophagectomy is particularly beneficial to reduce the incidence of postoperative respiratory complications, but may be associated with a higher incidence of left recurrent nerve palsy according to one large Japanese study [6]. In this study, we present our lymph node dissection technique, with specific attention to the micro-anatomical layer to avoid recurrent laryngeal nerve injury.

## Materials and methods

### Study design and patient characteristics

We previously reported our procedure and outcomes of thoracoscopic esophagectomy in the left lateral decubitus position [5] and have performed over 1100 minimally invasive esophagectomy procedures over the last 21 years. From May 2016 to December 2018, thoracoscopic resection for patients with esophageal cancer in the left lateral decubitus position was attempted in 87 patients by the same surgical team at Showa University Hospital. Inclusion criteria were patients with carcinoma of the thoracic esophagus, without serious cardiac or respiratory disease that would preclude safe conduct of surgery under general anesthesia, without metastases to other organs such as the lung or liver, and tumor stage lower than Stage T4b. Clinicopathological factors were classified according to the UICC-TNM (7th edition) criteria [7]. Surgical outcomes data were collected, and use of these data was approved by the Institutional Review Board of Showa University, Tokyo, Japan.

### Surgical procedure

#### Anesthesia, positioning, and port placement

**Fig. 1:**
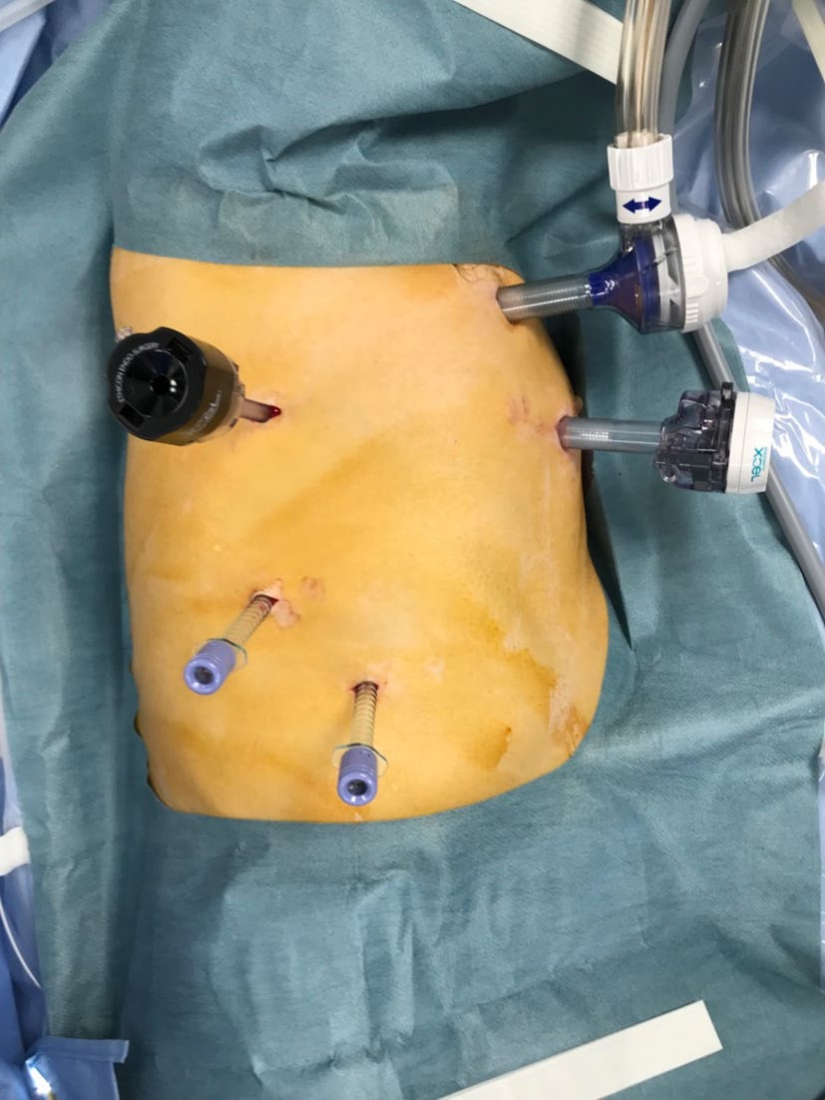
Port placement: three 5-mm ports and two 12-mm ports are used

Surgery was performed after induction of general anesthesia. In general, two-lung ventilation was used during the thoracoscopic and abdominal portions of the procedure. One-lung pulmonary ventilation with an 8-Fr spiral tube was occasionally used, and the right bronchus was occluded when two-lung ventilation was difficult. The thoracic portion of the operation was performed in the left lateral decubitus position with 15° head elevation. The surgeon stood on the dorsal side of the patient, and a high-definition video monitor was placed at the patient’s head. The first observation port (12 mm) was placed at the 5th intercostal space in the anterior axillary line. Carbon dioxide pneumothorax was achieved at a pressure of 8 mm Hg, and the right lung was decompressed. This was followed by placement of 5-mm ports for the operator in the fifth and eighth intercostal spaces at the posterior axillary line. A 5-mm port for the thoracoscope was inserted into the eighth intercostal region at the mid-axillary line, and 12-mm ports for the assistant were inserted in a slightly ventral position in the third intercostal space at the anterior axillary line (Fig. 1).

### Dissection technique

#### Right recurrent laryngeal nerve lymph node

**Fig. 2:**
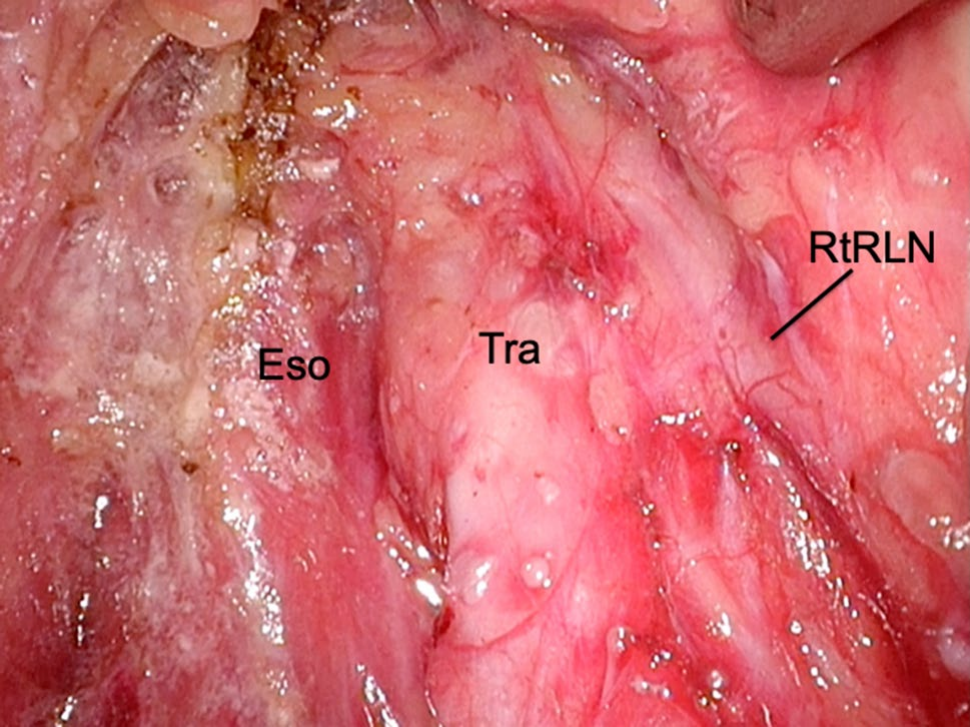
View after dissection around the right recurrent laryngeal nerve lymph node: we preserve the thin layer of connective tissue around the right recurrent laryngeal nerve. *Eso* esophagus, *RtRLN* right recurrent laryngeal nerve, *Tra* trachea

The lymph nodes around the right recurrent laryngeal nerve were resected. Since three to four branches run from the right recurrent laryngeal nerve toward the esophagus, these were divided with an ultrasonic device (HARMONIC ACE+7, Johnson and Johnson, New Brunswick, NJ, USA). In this dissection, we preserve the thin layer of connective tissue around the right recurrent laryngeal nerve, paying careful attention to the risk of thermal damage and avoiding traction on the nerve when dissecting the right recurrent laryngeal nerve lymph node (Fig. 2). We also preserve the thin native tissue overlaying the right recurrent laryngeal nerve.

#### Left recurrent laryngeal nerve lymph node on the dorsal side of the trachea

**Fig. 3:**
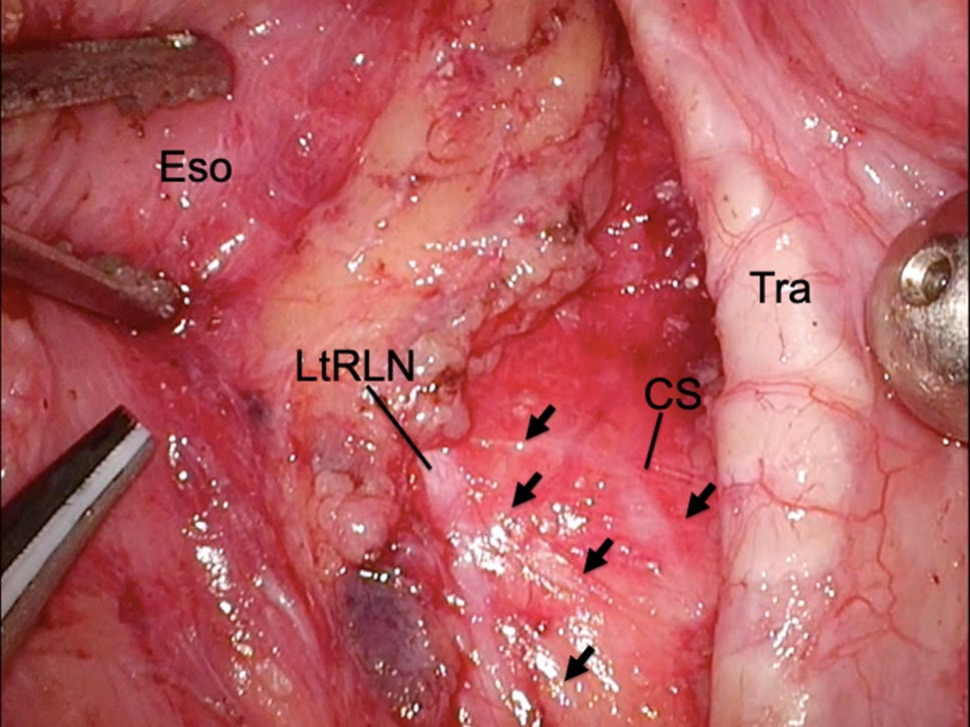
Dissection view around the left recurrent laryngeal nerve and cardiac branch of the sympathetic nerve: we dissect the ventral side with fatty tissue including the left recurrent laryngeal nerve and lymph nodes until we can confirm the overlying shiny thin layer of connective tissue. This shiny thin layer is on the cardiac branch of the sympathetic nerve and is preserved to avoid cardiac arrhythmias. Black arrows show the shiny thin layer. *CS* cardiac branch of the sympathetic nerve, *Eso* esophagus, *LtRLN* left recurrent laryngeal nerve, *Tra* trachea

We dissected the area between the trachea and esophagus using an ultrasonic instrument. First, we dissected the ventral side with adipose tissue including the left recurrent laryngeal nerve and lymph nodes until we confirmed the overlying shiny thin layer of connective tissue. This shiny thin layer is on the cardiac branch of the sympathetic nerve and is preserved to avoid cardiac arrhythmias (Fig. 3). After dissection inferior to the aortic arch to the cervicothoracic junction, we dissected around the proximal thoracic esophagus, and the esophagus was transected using an automatic suture device (Echelon Gold 60 mm; Johnson and Johnson). By rotating the trachea toward the ventral side, we obtained a good view of the adipose tissue, which contains the left recurrent laryngeal nerve and lymph nodes, without the esophagus.

#### Native tissue preservation technique of the recurrent laryngeal nerve

**Fig. 4:**
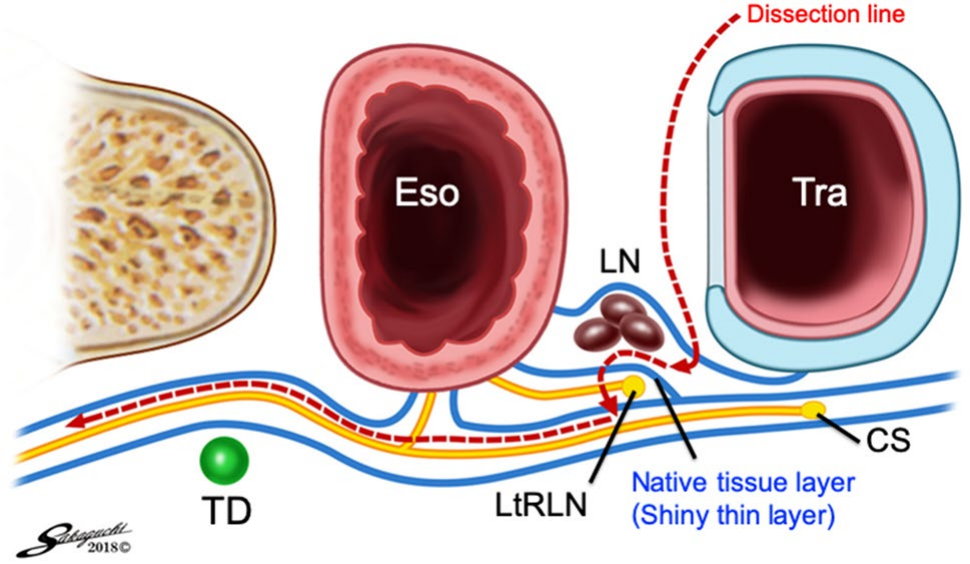
Coronal view of the dissection field: first, we dissect the dorsal side of the trachea. Then, the left recurrent laryngeal lymph nodes and adipose tissue are rotated counterclockwise from the ventral side to the dorsal side about the axis of the left recurrent laryngeal nerve. The blue line shows the native tissue layer (shiny thin layer) located above the left recurrent laryngeal and the cardiac branch of the sympathetic nerve. *Eso* esophagus, *CS* cardiac branch of sympathetic nerve, *LN* lymph node, *LtRLN* left recurrent laryngeal nerve, *TD* thoracic duct, *Tra* trachea (Color figure online)

**Fig. 5:**
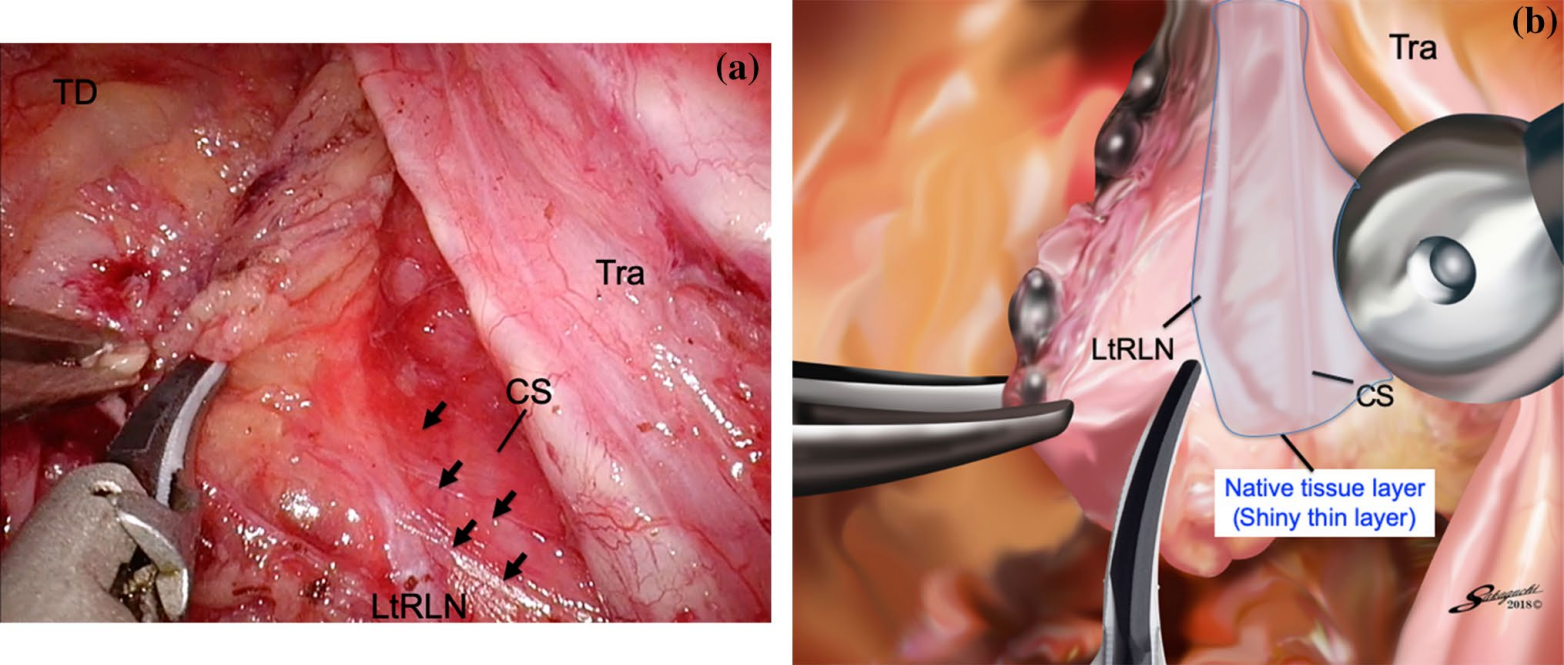
**A** Dissection view around the left recurrent laryngeal nerve and cardiac branch of the sympathetic nerve without showing the esophagus: the overlying thin layer of native tissue is identified, and the lymph nodes and fatty tissue are rotated counterclockwise from the ventral side to the dorsal side about the axis of the left recurrent laryngeal nerve. Black arrows show the shiny thin layer. **B** The left recurrent laryngeal nerve and lymph node are observed clearly after esophageal dissection. The native tissue layer (shiny thin layer) is observed above the left recurrent laryngeal nerve and cardiac branch of the sympathetic nerve (blue area); we should preserve this layer. *LtRLN* left recurrent laryngeal nerve, *CS* cardiac branch of the sympathetic nerve, *TD* thoracic duct, *Tra* trachea (Color figure online)

**Fig. 6:**
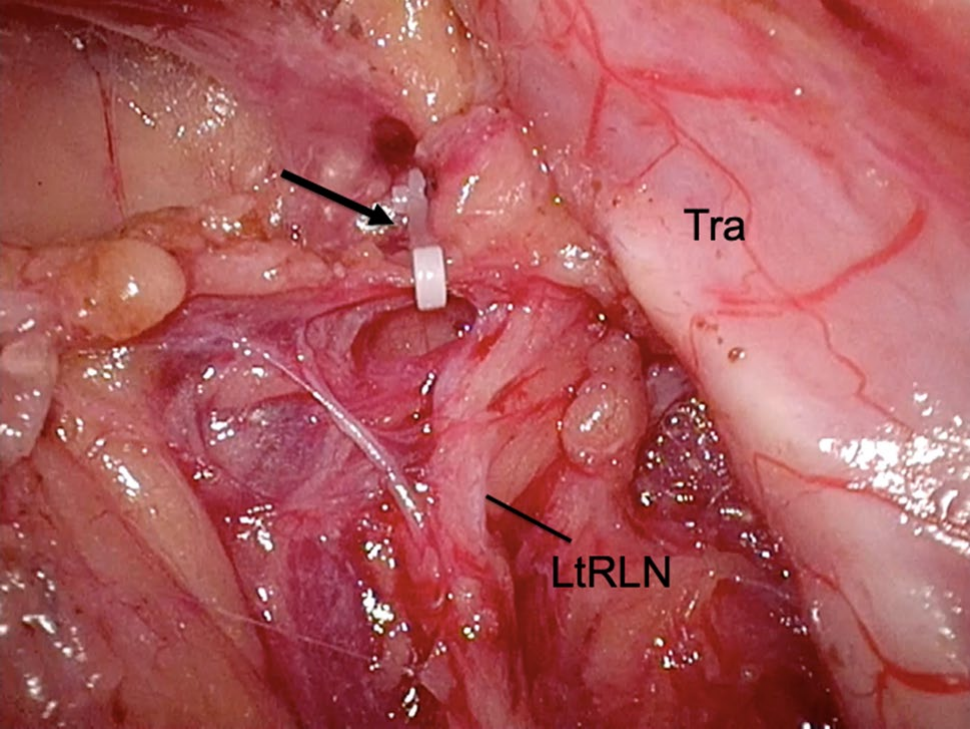
Dissection view of the superior side around the left recurrent laryngeal nerve. A clip (arrow) is placed to facilitate confirmation of the location during the cervical procedure. *LtRLN* left recurrent laryngeal nerve, *Tra* trachea

**Fig. 7:**
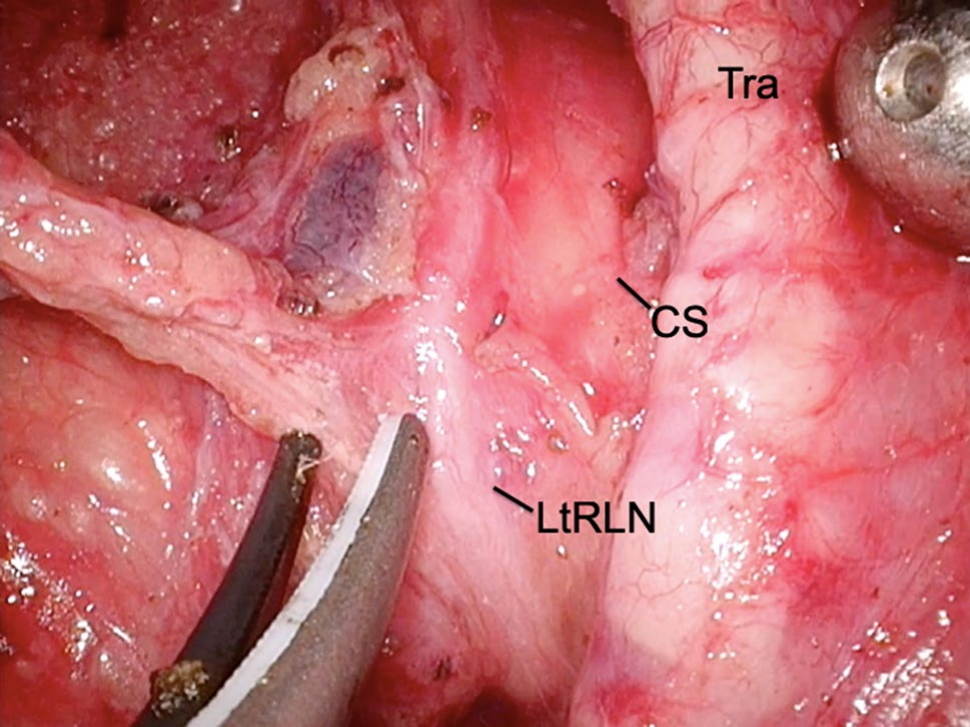
Native tissue preservation technique*:* the left recurrent laryngeal nerve lymph nodes are safely dissected after rotation along the dorsal side of the left recurrent laryngeal nerve with an adequate margin between the area of dissection and the nerve without touching and stretching the nerve. *CS* cardiac branch of the sympathetic nerve, *LtRLN* left recurrent laryngeal nerve, *Tra* trachea

**Fig. 8:**
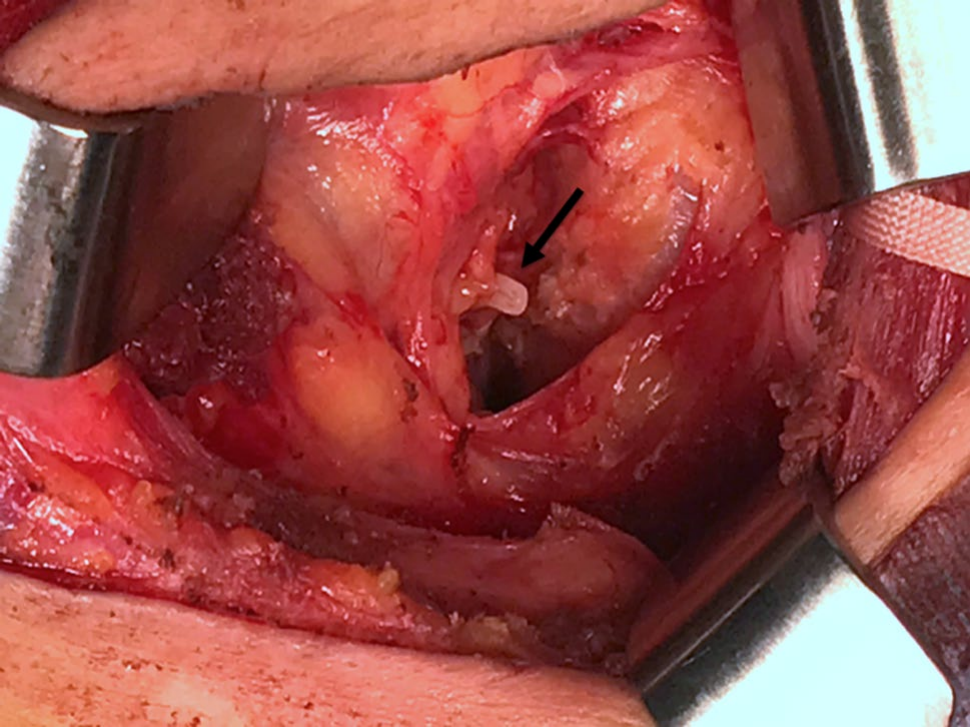
Cervical procedure view: intrathoracic superior landmark clip of the left recurrent laryngeal nerve lymph node facilitate confirmation of its location during cervical procedure (black arrow)

The aim of this technique is to prevent RLNP based on the premise that by not actually touching the nerve, traction and injury to the recurrent laryngeal nerve are limited while maintaining the normal anatomical position of the left recurrent laryngeal nerve. We rotate the left recurrent laryngeal lymph nodes and adipose tissue counterclockwise from the ventral side to the dorsal side about the axis of the left recurrent laryngeal nerve (Fig. 4). By preserving the thin native tissue layer around the left recurrent laryngeal nerve, traction and bending are avoided (Fig. 5A, B). We carefully avoid grasping the adipose tissue, avoiding traction on the nerve, and not touching the nerve when dissecting around the left recurrent laryngeal nerve lymph nodes. We mainly use an ultrasonic dissector, avoiding thermal damage while dissecting around the left recurrent laryngeal nerve. We limit the time of application of the ultrasonic instrument and use a short pitch dissection technique, limited to 1–2 s. Division of a few transverse vessels around the left recurrent laryngeal nerve was confirmed after ultrasonic dissection. Recently, we have used the HARMONIC HD 1000i Shears (Johnson and Johnson) during minimally invasive esophagectomy, which allows dissection without using scissors around the recurrent laryngeal nerve and its branches. After the adipose tissue and lymph nodes are rotated dorsally to the left recurrent laryngeal nerve, we confirm the location of the left recurrent laryngeal nerve under the thin layer of connective tissue. The overlying adipose tissue and lymph nodes on the superior aspect are dissected, and the area marked with a clip to facilitate confirmation of the location during the cervical procedure (Fig. 6). These lymph nodes were dissected from caudal to cranial because there are small branches of the left recurrent laryngeal nerve passing from caudal to cranial, and these branches should be divided in the same direction to avoid injury to the main nerve. We can then safely dissect the left recurrent laryngeal nerve lymph nodes that are already rotated toward the dorsal side of the left recurrent laryngeal nerve with an adequate margin from the nerve (Fig. 7). The left recurrent laryngeal nerve lymph nodes are removed while maintaining the normal anatomical position of the left recurrent laryngeal nerve without touching and stretching the nerve (video). The intrathoracic superior landmark clip placed on the left recurrent laryngeal nerve lymph node can help confirm the location of the lymph node and provide information on how much of the lymph node can be dissected during the cervical procedure (Fig. 8).

#### Evaluation for RLNP

The presence of RLNP was assessed by the anesthesiologist at extubation and assessed by an endoscopist and an otolaryngologist using a flexible laryngoscope before starting oral intake (postoperative day 5) regardless of the presence or absence of hoarseness or paralysis. We also evaluate all patients for hoarseness until discharge. The grade of RLNP was classified according to the Clavien–Dindo classification [8] and supplementary criteria advocated by the Japan Clinical Oncology Group [9]. The definition of RLNP was any classification higher than grade II. If a patient was found to have RLNP, an otolaryngologist performed a follow-up examination. After discharge, patients were continuously followed up regarding hoarseness of their voice once every month in the first 6 months and every 3 months thereafter at the outpatient clinic. These data were collected by review of medical records.

## Results

Demographic characteristics for all patients are shown in Table 1. The mean age was 64.9 years and the study group included 75% men. Tumors were located in the cervical (2.3%), upper thoracic (14%), middle thoracic (46%), lower thoracic (29%), and abdominal (9.2%) esophagus. Squamous cell carcinoma was diagnosed in 87%, adenocarcinoma in 9.2%, and carcinosarcoma in 2.3% of patients on the basis of histologic findings. Preoperative therapy was administered to 93% of patients. Neoadjuvant chemotherapy was administered to 89% of patients. Four patients (4.6%) with suspected cT4 lesions were administered neoadjuvant chemo-radiation therapy. Lung adhesions were noted during surgery in 32% of patients. Thoracoscopic esophagectomy was completed in all patients without conversion to open thoracotomy.

**Table 1:** Patient characteristics (*N* = 87)

Age, mean (years)	64.9
Male, *n* (%)	65 (75)
Tumor location	
Cervical esophagus, *n* (%)	2 (2.3)
Upper esophagus, *n* (%)	12 (14)
Middle esophagus, *n* (%)	40 (46)
Lower esophagus, *n* (%)	25 (29)
Abdominal esophagus, *n* (%)	8 (9.2)
Histologic findings	
Squamous cell carcinoma, *n* (%)	76 (87)
Adenocarcinoma, *n* (%)	8 (9.2)
Carcinosarcoma, *n* (%)	2 (2.3)
Neuroendocrine carcinoma	1 (1.1)
pTNM stage	
Stage IA, *n* (%)	28 (3.2)
Stage IB, *n* (%)	3 (3.4)
Stage IIA, *n* (%)	11 (13)
Stage IIB, *n* (%)	17 (19)
Stage IIIA, *n* (%)	15 (17)
Stage IIIB, *n* (%)	6 (6.9)
Stage IIIC, *n* (%)	7 (8.0)
Stage IV, *n* (%)	0 (0)
Neoadjuvant therapy	
None, *n* (%)	6 (6.9)
Chemotherapy, *n* (%)	77 (89)
Chemo-radiation, *n* (%)	4 (4.6)
Pleural adhesions	
Yes, *n* (%)	28 (32)
No, *n* (%)	59 (68)
Number of lymphadenectomy fields	
Two, *n* (%)	38 (44)
Three, *n* (%)	49 (56)
Anastomosis site	
Cervical, *n* (%)	86 (99)
Intrathoracic, *n* (%)	1 (1.8)
Conversion to thoracotomy, *n* (%)	0 (0)

Surgical outcomes and complications are shown in Table 2. There were no blood transfusions due to thoracoscopic blood loss. The mean number of thoracic lymph nodes retrieved was 26.5, and the mean number for all lymph nodes was 58.6. The overall complication rate was 20% including anastomotic leak in 3.4% and postoperative bleeding in 2.3% of cases. There were no instances of grade II or higher postoperative RLNP (Clavien–Dindo classification). However, temporary hoarseness (Clavien–Dindo grade I) was observed in two patients (2.3%) who recovered without specific therapy within 2 months. Arrhythmias were observed in 4.6% of patients, pulmonary thrombosis in 1.1%, atelectasis in 1.1%, and pneumonia in 3.4%. Reoperation within 30 days was necessary in 2.3% of patients due to postoperative bleeding. There was no mortality within 30 days.

**Table 2:** Surgical outcomes and postoperative complications (n = 87)

Thoracic operative time (min), mean	183.7
Thoracic blood loss (ml), mean	57.6
Blood transfusion (ml), mean	0
Extubation (days after surgery), mean	0
Postoperative length of stay (days), mean	16.3
Number of retrieved thoracic lymph nodes, mean	26.5
Number of retrieved total lymph nodes, mean	58.6
Overall complication rate, *n* (%)	18 (20)
Surgical complications	
Chylothorax, *n* (%)	0 (0)
Anastomotic leakage, *n* (%)	3 (3.4)
Postoperative bleeding, *n* (%)	2 (2.3)
Recurrent laryngeal nerve paralysis, *n* (%)	0 (0)
Non-surgical complications	
Arrhythmia, *n* (%)	4 (4.6)
Pneumonia, *n* (%)	3 (3.4)
Pulmonary thrombosis, *n* (%)	1 (1.1)
Atelectasis, *n* (%)	1 (1.1)
Reoperation within 30 days, *n* (%)	2 (2.3)
Mortality within 30 days, *n* (%)	0 (0)

## Discussion

By avoiding direct nerve injury, unreasonable traction, thermal damage, and other factors associated with RLNP, the incidence of RLNP should be minimized. Herein, we have reviewed the dissection techniques used to limit the occurrence of RLNP, with specific focus on the anatomical layer around the recurrent laryngeal nerve. This new technique for limiting the incidence of RLNP is based on the simple concept of preserving the thin layer of tissue on the left recurrent laryngeal nerve. We refer to this as the *native tissue preservation* technique. Although we dissected the recurrent laryngeal nerve lymph nodes bilaterally, we did not note any incidence of RLNP (Clavien-Dindo classification > grade II) in this series of patients. This novel technique enabled an extended radical lymphadenectomy with retrieval of a mean of 26.5 lymph nodes from the mediastinum and 58.6 total lymph nodes.

Thoracic esophageal cancer accounts for approximately 90% of esophageal cancers in Japan, with a relatively high risk of metastases to upper mediastinal lymph nodes along the recurrent laryngeal nerves bilaterally [10]. Moreover, we clinically suspected metastasis in the recurrent laryngeal nerve lymph nodes in 51 cases (58.6%). Right recurrent laryngeal nerve lymph node metastasis was noted in 47 cases (54%) and left recurrent laryngeal nerve lymph node metastasis was noted in 19 cases (21.8%); bilateral recurrent laryngeal nerve lymph node metastasis was observed in 15 cases (17.2%) in our study. We chose neoadjuvant chemotherapy for 89% cases in this study, and a total of 24 cases (27.6%) of lymph node metastasis around the recurrent laryngeal nerve was diagnosed. Based on the pathological findings, lymph node metastasis on the right side was detected in 18 cases (20.7%), while lymph node metastasis on the left side was detected in 12 cases (13.8%); bilateral metastasis was detected in 6 cases (6.9%). Given the nature of the disease, extended radical lymphadenectomy including the upper mediastinal lymph nodes along both recurrent laryngeal nerves is considered a standard surgical approach in Japan [11–13]. Although it depends on the extent of lymph node dissection performed, the incidence of RLNP after esophagectomy with radical lymph node dissection is reported to range from 8.3 to 40.9% [2–4]. This rate of RLNP after a three-field lymph node dissection is unacceptably high following esophagectomy. Radical lymphadenectomy including the upper mediastinal nodes after neoadjuvant chemotherapy for patients with advanced esophageal cancer improved the overall 5-year survival rate from 50 to 70% in our institution [5].

The resection of esophageal cancer has recently been performed thoracoscopically for many patients [6]. We use thoracoscopic magnification during lymph node dissection and divide the branches of the recurrent laryngeal nerve carefully to avoid thermal damage, stretching, cutting, compression, and vascular compromise. Placing a tape around the recurrent laryngeal nerve during lymphadenectomy is usually performed during radical esophagectomy performed through a thoracotomy [11, 13]. However, minimally invasive esophagectomy has a higher rate of recurrent nerve palsy compared with open surgery (10.3% vs. 8.1%), suggesting the need to more carefully avoid RLNP when performing resection of esophageal cancer [6]. Minimally invasive esophagectomy has good outcomes usually with little bleeding, but we noticed thermal damage and stretching of the recurrent laryngeal nerve during the lymphadenectomy. We believe these factors contribute to the development of RLNP and care must be taken to avoid these actions intraoperatively.

Most dissections were performed with an ultrasonic surgical device when performing the upper mediastinal lymphadenectomy along the recurrent laryngeal nerves during minimally invasive esophagectomy. Special care is taken to avoid thermal damage and cavitation. There is evidence that the use of electrosurgical devices may lead to inadvertent damage to nearby structures such as the bowel, nerves, or blood vessels through the lateral spread of thermal energy [14–17]. Studies also show a correlation of the degree of thermal injury with lateral thermal spread [18, 19]. A study of the Harmonic Scalpel device demonstrated that it may be used without a substantial rise in the temperature of adjacent tissues. An increase in tissue temperature above 42 °C results in both damage to cell membranes and protein denaturation [20]. Sutton et al. suggested that the use of electrosurgical instruments is associated with a significant rise in temperature at the tip of the instrument, proportional to the power and length of time of application [21]. We are vigilant regarding the duration of application and use the short pitch dissection technique within 1–2 s when dissecting connective tissue and small vessels. These precautions may limit thermal damage to the recurrent laryngeal nerve.

To investigate the influence of distance from the instrument on damage to the recurrent laryngeal nerve, Megan et al. compared thermal spread and recurrent laryngeal nerve function with the THUNDERBEAT Open Fine Jaw device, the bipolar Ligasure Small Jaw, and the ultrasonic Harmonic Focus for open thyroidectomy [22]. This study concluded that RLN injury did not occur if the devices were used approximately 2 mm away from the nerve. We did not maintain this 2 mm margin from the nerve because of existing adipose and lymph node tissue that must be radically resected inside that margin. Although we cannot conclude a specific safe distance from the nerve based on this study, we believe that attention to meticulous dissection during lymphadenectomy around the nerve with magnification can avoid RLNP due to thermal damage.

Stretching of the recurrent laryngeal nerve during lymphadenectomy must be avoided. We believe that excessive stretching is the most common mechanism of recurrent laryngeal nerve injury. The left recurrent laryngeal nerve is more frequently involved, probably because the longer course of the nerve creates additional vulnerability, especially within the mediastinum. However, this native tissue preservation technique was used around not only the left recurrent laryngeal nerve area but also the right recurrent laryngeal nerve lymph node. Thyroid surgeons suggest that excessive traction is the most common mechanism of recurrent laryngeal nerve injury and suggest that intraoperative nerve monitoring may be a useful adjunct to decrease the rate of injury [23–25]. We used intraoperative nerve monitoring in the past, but it was difficult to prevent injury because the monitoring was not continuous. We could only determine the incidence of recurrent laryngeal nerve palsy, but continuous intraoperative nerve monitoring was useful to decrease the injury in thyroid surgery [24]. In a recent animal study, Deguchi et al. studied approaches to decrease the incidence of RLN damage during esophageal surgery [26] and suggested that continuous intraoperative nerve monitoring may be useful to decrease the incidence of nerve injury in esophageal surgery. The underlying concept of this native tissue preservation technique is to maintain the recurrent laryngeal nerve in its normal anatomical position without stretching, thermal damage, or touching the nerve, and this approach limits the incidence of RLNP, as there was no incidence of RLNP in 87 consecutive patients in this study. The native tissue preservation technique also limits the incidence of vascular compromise to the recurrent laryngeal nerve. Blood flow affects nerve function and regeneration [27, 28]. We believe that the thin layer of connective tissue around the recurrent laryngeal nerve also preserves the small blood vessels that supply the recurrent laryngeal nerve.

Although it has been suggested that neoadjuvant chemo-radiation therapy increases postoperative complications and mortality compared to surgery alone [29], we performed neoadjuvant chemo-radiation (40 Gy) followed by minimally invasive esophagectomy using the native tissue preservation technique after 4 weeks in four cases. We did not note a significant difference in the thoracic operation time (175.5 min), thoracic blood loss (68 ml), and postoperative length of stay (16.5 days) compared to the other techniques. There were no complications in our patients. Therefore, our procedure can be performed safely for patients who receive neoadjuvant chemo-radiation therapy.

Postoperative bleeding was observed in two patients (2.3%) in this study. One patient showed bleeding from a branch of the right bronchial artery on postoperative day 2 and required reoperation. Another patient presented with postoperative bleeding from a branch of the right gastroepiploic artery after gastric tube reconstruction on postoperative day 0 and required reoperation. These bleeding events were not related to lymphadenectomy, and the patients were discharged following an extended standard perioperative course. Pneumonia was observed in three patients (3.4%) in this study, which is a lower incidence than previously reported [2, 3, 6]. Among these three patients, one patient had previous experienced postoperative bleeding, and he developed atelectasis, anastomotic leakage, and pneumonia. Two elderly patients (over 85 years old) developed aspiration pneumonia, which suggests that this technique may limit the development of aspiration pneumonia. We believe that this approach helps limit the incidence of recurrent laryngeal nerve palsy and is a simple technique based on anatomical structures, facilitating its use by most esophageal surgeons.

This study has limitations. It is a single-institution retrospective study, and all operations were performed by a highly experienced team. These results may not be generalizable but are encouraging and support further study of the native tissue preservation technique. These results are expected to contribute to the prevention of recurrent laryngeal nerve palsy during minimally invasive esophagectomy with radical lymph node dissection.

In conclusion, the native tissue preservation technique maintains the normal anatomical position of the recurrent laryngeal nerve, avoiding stretching, thermal damage, and direct contact to the nerve. This technique was used in 87 consecutive patients who underwent minimally invasive radical esophagectomy with radical lymph node dissection, and there was no incidence of RLNP. This native tissue preservation technique may reduce the incidence of recurrent laryngeal nerve paralysis after minimally invasive esophagectomy with radical lymph node dissection.

## Electronic supplementary material

Below is the link to the electronic supplementary material.

Supplementary file1 (M4V 327204 kb)
